# Advancing Circular Composite Strategies by Vitrimer-Enabled Reuse of Unidirectional Laminates

**DOI:** 10.3390/polym18020300

**Published:** 2026-01-22

**Authors:** Jannick Fuchs, Nico Schuhmann, Jonathan Alms, Christian Hopmann

**Affiliations:** Institute for Plastics Processing in Industry and Crafts, RWTH Aachen University, 52074 Aachen, Germany

**Keywords:** carbon fibre, fracture toughness, recycling, surface roughness, vitrimer, vitrimeric resin

## Abstract

To efficiently reuse endless fibre-reinforced composites after their life cycle, the recovery of endless fibres including matrix material with subsequent reprocessing in their original state is desirable. Thanks to their covalent adaptive networks, vitrimers offer ideal properties for enabling new repair and circular strategies for composites. In order to evaluate the detachability—meaning the separation of single laminate layers—and recycling potential for continuous fibre reinforcement, process routes and quality parameters must be established. In this study, the double cantilever beam test is used to test the adhesion based on the detachment of continuous fibre layers, and the interlaminare fracture toughness of mode I (G_IC_) is measured as a parameter for the required energy for detachment. It was shown that G_IC_ increases above the vitrimer transition temperature and is higher than for reference specimens with an epoxy matrix. Surface roughness is measured to determine the mechanical and thermal degradation of the chemical network structure and additionally shows fibre cracking and defects in fibre–matrix interfaces. This allows the recycling process to be evaluated up to the production of a second generation, with the aim of identifying the recycling potential of the vitrimer matrix and implementing it for industrial processes. An efficient recycling strategy of the continuous fibre-reinforced vitrimers was thus demonstrated by hot pressing at 190 °C for 45 min, giving vitrimer samples a second life.

## 1. Introduction

Continuous fibre-reinforced plastics (FRPs) are increasingly being used for structural lightweight construction in many applications in the renewable energy, transport, mobility, and aviation sectors. Applications range from wind turbine blades and type-IV hydrogen pressure vessels to aircraft wings [1]. Due to their outstanding weight-specific stiffness and strength, carbon fibre-reinforced plastics (CFRPs) in particular are used in high-performance applications. The matrix material fulfilling these demanding applications comprises various thermosets that are explicitly tailored to the respective composite manufacturing processes [2]. Thermosets are more temperature-resistant due to covalent cross-linked networks and have a higher creep resistance than thermoplastic matrices [3,4]. The biggest disadvantage of thermosets is poor recyclability, as they can no longer be melted after cross-linking [5,6].

As carbon fibre is a very energy-intensive product due its manufacturing process, it is very important to recover the fibre as gently as possible with minimal fibre length loss or to find subsequent uses for the products [5,6]. Even better is extending the service life of FRP components or directly preserving and reusing the configuration of endless fibre reinforcement [7]. For wet filament winding, it is desirable to recover unwound rovings after the use phase of the component. A novel process was patented by Henning and Jongebloed for thermoplastic materials with lesser strength and stiffness in comparison to thermoset materials, as well as significant damage to the material after processing and unwinding the rovings [8,9].

Likewise, suitable processes must be developed for the purpose of sustainable composite manufacturing. Existing recycling approaches for FRPs offer opportunities but also present some obstacles. Mechanical recycling shortens the fibre length and thus reduces the mechanical properties of the recyclate [10], pyrolysis completely degrades the matrix and can damage the fibre surface, and solvolysis is costly due to the subsequent separation of the matrix from the solution and removes the sizing from the fibre [5,6]. The use of the innovative new class of vitrimers can enable new process routes to directly reuse continuous fibre reinforcement through covalent adaptive networks (CANs) [11]. The presented study aims to develop a processing route in which continuous unidirectional carbon fibre reinforcement can be detached in separate laminate layers and reused in similar applications without additional recycling steps using vitrimeric resin.

### Vitrimeric Resin as Reusable Matrix System

Vitrimers have been introduced as a promising alternative to conventional thermosets, offering potential for repair, reuse, and recycling (3R). They feature dynamic covalent bonds that form a dynamic CAN during curing, enabling reconfiguration of the polymer network in a fully cured state. This property opens possibilities for the mechanical recycling of thermosetting materials as well as fibre recovery [12,13,14]. The topology freezing temperature (T_v_) defines the transition of vitrimers from a solid-like to a viscoelastic liquid state, governed by the onset of dynamic covalent bond exchange [15]. T_v_ is determined using either mechanical testing or rheology, examining stress relaxation, creep, elongational creep [16], and thermomechanical analysis [14,17]; however, these methods are highly sensitive to applied stress, catalyst concentration, and filler content, which can lead to apparent shifts in T_v_ [15]. At T_v_, vitrimers exhibit a pronounced drop in viscosity, enhanced flow, and a transition to malleable and reprocessable states [15,18]. Despite advances in characterisation, no universal, direct, and fully stress-free method for determining T_v_ has yet been established.

With their chemical properties, vitrimers allow for reshaping, welding, and repair [11,13,19], as well as chemical dissolution of the matrix under comparatively mild conditions (typically 60–160 °C in alcohols, glycols, or amine media) [20]. These routes enable the recovery of essentially undamaged carbon fibres and, in many systems, the closed-loop reuse of matrix oligomers to regenerate vitrimer resins with a ≥90–98% retention of thermomechanical properties [21]. Multi-cycle reprocessing with limited strength loss further extends component lifetimes prior to end-of-life recycling [22]. However, deployment is still constrained by challenges in scaling matrix reprocessing beyond lab-scale coupons [23]: the separation and purification of degradation products, process kinetics and energy inputs, and the economics of closed-loop operation remain insufficient [24]. Thus, while vitrimer chemistry clearly enables recyclable matrices and repeated reprocessing [21,22], the critical next steps lie in industrial implementation and process integration at the structural scale. This study aims to understand reprocessing and its influence on composite properties. Fibre-reinforced components with a vitrimer matrix are typically manufactured by hot pressing imine- and ester-based vitrimers or by compressing resin transfer moulding and performing vacuum-assisted resin infusion of disulfide-crosslinked epoxy vitrimers [25,26].

The disulfide-crosslinked epoxy vitrimers used in this study employ the curing agent 4-AFD, which contains dynamic molecular bonds (disulfide bridges) but reacts with the epoxy resin through a catalyst-free amine reaction consistent with conventional curing mechanisms [13]. The compatibility of Bis-(4-Aminophenyl-)Disulfide (4-AFD) with standard epoxy resins and the corresponding stoichiometry has been investigated in earlier studies and is reproduced here [12,13]. Material processing in this study focuses on vacuum-assisted resin infusion (VARI), chosen for its high reproducibility due to controlled processing parameters, whereas previous studies primarily emphasised wet filament winding. Hot pressing has been employed for the reconfiguration of endless carbon fibre-reinforced vitrimers (CFRVs) [12,13].

The double cantilever beam (DCB) test is a method of investigating the fatigue or fracture mechanics of composite specimens [27]. The assessment of reusability in unidirectional reinforced vitrimers is anchored within this mechanical test, which, through methodological innovations and complementary mechanical testing, provides robust insights into the interfacial fracture toughness and healing efficiency of CFRVs [22,28,29]. This is a key benefit in comparison to epoxy resin testing. However, significant challenges remain in comprehending the mechanistic underpinnings of bond exchange dynamics and in optimising material design for sustainable, scalable applications. Moreover, current studies have focused on the investigation of ester-based vitrimers or glass fibre reinforcement [29] to enable microscopic or X-ray computed tomography examinations [28]. The objective of this study is to address the disparity between materials derived from disulfide exchange cross-linking and carbon fibre, which are utilised in lightweight structural construction, such as resin transfer moulding or wet filament winding.

## 2. Materials and Methods

**Figure 1:**
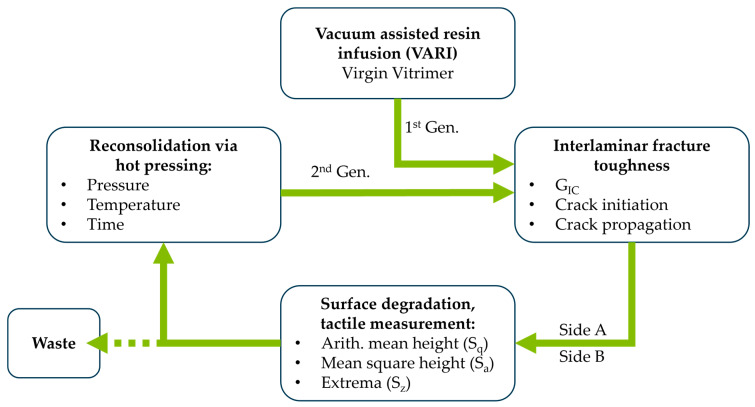
Approach of the presented study to determine mechanical properties and specimen degradation over multiple generations of CFRVs.

Figure 1 shows the approach of the presented study to manufacturing CFRVs and the subsequent steps to determining the recyclability with unidirectional reinforcement, as well as surface degradation via tactile measurements. The specimen will then be reconsolidated as a 2nd generation via a newly developed hot-pressing cycle for vitrimeric resin. The materials and methods used in the study are described below, according to this approach.

The material used in the presented study is epoxy resin EPON 0162 by Westlake Epoxy GmbH, Duisburg, Germany, with 4-AFD in a weight ratio of 100:43. Both materials were preheated in an oven at 80 °C to ensure a sufficient low mixing viscosity and were afterwards mixed and degassed under vacuum conditions. To manufacture the CFRV specimen of the 1st generation, vacuum-assisted resin infusion (VARI) was performed in an autoclave, Scholz Maschinenbau GmbH & Co. KG, Coesfeld, Germany. Thereafter, a glass plate and six layers of unidirectional carbon fibre K-UD-270 from Haufler Composites GmbH & Co. Kg, Blaubeuren, Germany, with an area weight of 270 g/m^2^ were preheated to 90 °C. To ensure that an initial crack formed between the top three and bottom three layers, a heat-resistant polytetraflourethylene film with a thickness of 25 µm was inserted with a depth of 50 mm into the centre of the test specimen. The vitrimeric resin was infused with a vacuum of 130 mbar into the laminate and was afterwards cured for 1 h at 90 °C and 2 h at 140 °C with a constant pressure of 2 bar. As stated in preceding studies by Lorenz et al., material characterisation for rheological behaviour, cure cycle, and the full degree of cure of the vitrimeric resin was conducted [13]. T_g_, as determined by dynamic scanning calorimetry, was 134 °C, while T_v_ was previously determined by thermomechanical analysis to be below this temperature, at 110 °C. Therefore, a temperature of 120 °C was used in this study, as this is in between T_v_ and T_g_.

The infused specimens of the 1st generation had a thickness of 2 mm and were cut to dimensions of 5 × 30 mm^2^ using a diamond circular saw. An average fibre volume content of 56.89 ± 2.77% was determined from five representative microsections. To produce reference specimens with conventional curing systems, the resin EPON 0162, commercial hardener EPIKURE 04976, and EPIKOTE catalyst 04976, both supplied by Westlake Epoxy GmbH, Duisburg, Germany, with a weight ratio of 100:80:1.5, were used to manufacture specimens. Identical process parameters were used during VARI to ensure comparability.

**Figure 2:**
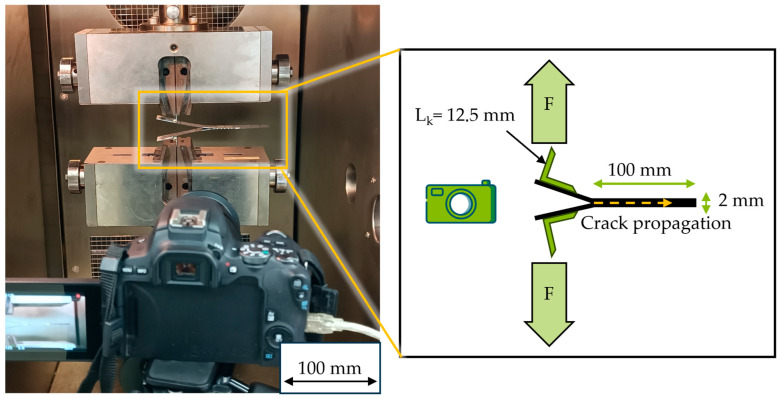
Test setup to record crack propagation, marked with a dotted arrow, with the DCB to analyse interlaminar fracture toughness.

To determine the interlaminar fracture toughness according to mode I failure behaviour, where force acts perpendicular to the crack opening, double cantilever beam tests (DCB) were performed using the universal testing machine Z150, ZwickRoell GmbH & Co. KG, Ulm, Germany, with two different load cells, with ZwickRoell Xforce K 150 kN for higher forces and ZwickRoell Xforce K 10 kN for lower displayed forces. For each combination of material and temperature, five specimens were tested. The specimens were glued to metal hinges (measuring 25 × 25 mm^2^) on both sides, using high-strength and temperature-resistant epoxy adhesive EA 9497, Loctite, Duesseldorf, Germany (Figure 2). This allowed for a clamping length (l_k_) of 12.5 mm. Crack propagation was monitored via markings with defined spacing on the surface of the specimen using video recording. The specimen was pulled apart at 2 mm/min. Test temperatures were varied between room temperatures (approx. 25 °C); below vitrification temperature (T_v_) was at 80 °C and above T_v_ at 120 °C using a climate chamber. The G_IC_ and the energy required for crack initiation were then calculated from the determined force–displacement curves according to Equation (1).

After separation of the specimen, the surface of both sides from the detached side of the laminates (A-side and B-side) was measured with the tactile roughness measurement device, Waveline W802R, Jenoptik AG, Jena, Germany. The measurement enables the profiling of surfaces with an accuracy of ±0.3 nm. To enable a surface comparison within and across generations, the same fracture surface measuring 4 × 4 mm^2^ was measured. A total of 5000 points per measuring line were recorded at a distance of 0.8 µm from each other at a speed of 50 mm/s.

After testing the specimens with vitrimeric resin, they were reconsolidated to form specimens of the second generation. Therefore, the cracked surfaces were draped on top of each other and were pressed in a tool using a stamp. Two screw clamps were tightened to 20 Nm of torque, resulting in an applied pressure of approximately 8–8.5 MPa. The surface was then reconsolidated at 190 °C for 45 min. Preliminary investigations have shown that these are suitable process parameters for healing the CFRV presented in this study [30]. Reconsolidated specimens were used to investigate the recyclability in the form of direct reprocessing of the CFRV as second-generation specimens, as shown in the schematic in Figure 1. In order to gain a more profound understanding of the fracture patterns, the fracture surfaces of the initiation and propagation zones of the crack were examined using field emission scanning electron microscopy (FESEM).

### Characterisation Methods for Assessing Reusability of Carbon Fibre Composites

There is no universal quality control for the recyclability of FRP. Often, the degree of fibre length reduction, the resulting mechanical properties in terms of stiffness and strength, or the CO_2_ equivalent of the recycling processes are evaluated [5,31]. In this study, the initial detachment behaviour is investigated using DCB in accordance with ISO 15024 [32]. This allows the interlaminar fracture toughness for unidirectional fibre-reinforced test specimens to be calculated according to Equation (1) [32]. This serves as an indication of how much energy must be expended to ensure recycling after the first manufacturing of the CFRV. Greater energy expenditure leads to a high risk of fibre matrix detachment, which could impede reconsolidation. To calculate G_IC_ in (1), the load (P) and the load in displacement (δ) are needed, as well as the specimen width (b), (initial) delamination length ($a_{0}$ and a), and the extrapolation of a linear fit through the data as an x-intercept (Δ) [32]:

(1) $$G_{IC}=\frac{3P\delta }{2ba\;+\;\left|∆\right|}\times F$$

With the correction for lever distance F, which takes the distance from the loading pin to the midplane of the half-beam ($l_{1}$) into account [32]:

(2) $$F=1\;-\;\frac{3}{10}\;\times \;{\left(\frac{\delta }{a}\right)}^{2}-\;\frac{3}{2}\;{\left(\frac{\delta l_{1}}{a2}\right)}^{2}$$

For Equations (1) and (2), the ratio of $\frac{\delta }{a}>0.4$, the correction factor in this study, is small, with F~0.98 for most of the conducted tests.

The degradation of the composite in between reuse cycles needs to be rethought, as there is no reduction in fibre length and the fibre coating or its sizing is not removed in the process. El Arwadi et al. report on the correlation between the self-healing properties of aromatic thermosetting copolyester and surface degradation after tearing the samples apart [33]. The surface roughness increased by an average of 4 µm after 4 healing cycles in comparison to the reference, and after just one cycle, the absolute average surface roughness increased up to 30 µm. Comparable to the method described, this study uses tactile measurements on the detached surfaces to demonstrate the self-healing ability of the vitrimer matrix after detaching and through reconsolidation. The transfer of El Arwadi et al.’s findings to vitrimers based on disulfide exchange could create comparability between material systems and enable the successful advancement of a continuous-fibre CFRV recycling strategy.

## 3. Results

In order to evaluate the recyclability of the CFRV, the G_IC_ is tested at various temperatures. There are three decisive temperature levels, which are as follows: temperature below the *T_v_*; the temperature between *T_v_* and the glass transition temperature (*T_g_*); and the temperature between *T_g_* and the matrix degradation temperature. It is expected that less force will be required to detach already cured unidirectional laminate layers at higher temperatures with a lower G_IC_, as the activation of the CAN above *T_v_* makes it easier to separate the interlaminate layers. However, increased temperatures significantly reduce the stiffness of fibre-reinforced specimens, particularly of the CFRV, which is why the DCB can only be performed at temperatures up to 120 °C for the vitrimer matrix, which excludes the temperature range between T_g_ and the degradation temperature.

**Figure 3:**
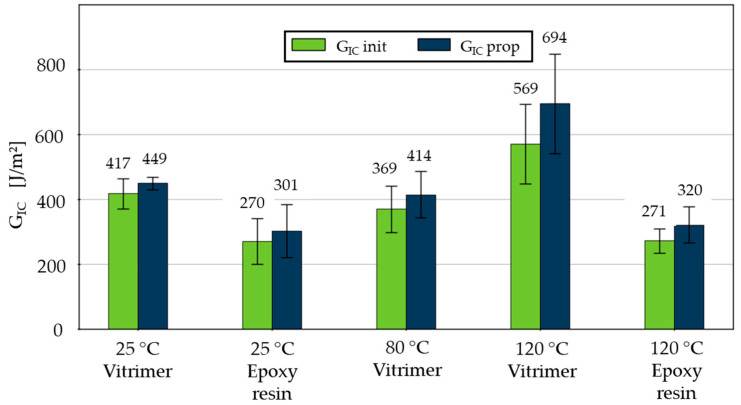
Comparison of G_IC_ initiation (init) and G_IC_ propagation (prop) for tested materials under different temperatures from first-generation specimen.

A general overview of the G_Ic_ of epoxy and vitrimer resin is given in Figure 3. The initiation energy release rate G_Ic_ init is defined as the energy release rate at the onset of crack growth from the precrack, whereas G_Ic_ prop describes the resistance during stable delamination growth and is typically higher due to fibre bridging effects. A general observation of the dataset from virgin samples indicates that, irrespective of the temperature, reduced G_Ic_ was documented for the detachment of epoxy resin during the crack initiation as well as crack propagation. This phenomenon appears to be almost constant, which is in contrast to the vitrimeric resin. Samples with a vitrified matrix demonstrate a higher G_Ic_, exhibiting a pronounced increase in conjunction with a high standard deviation occurring above T_v_. This shows that detaching CFRVs is temperature-dependent and can indicate the temperature range of T_v_ of the vitrimer in contrast to the epoxy resin. The subsequent section will discuss the material effects on measured G_Ic_, comparing epoxy and vitrimer resin.

### 3.1. Vitrimer Versus Epoxy Matrix

**Figure 4:**
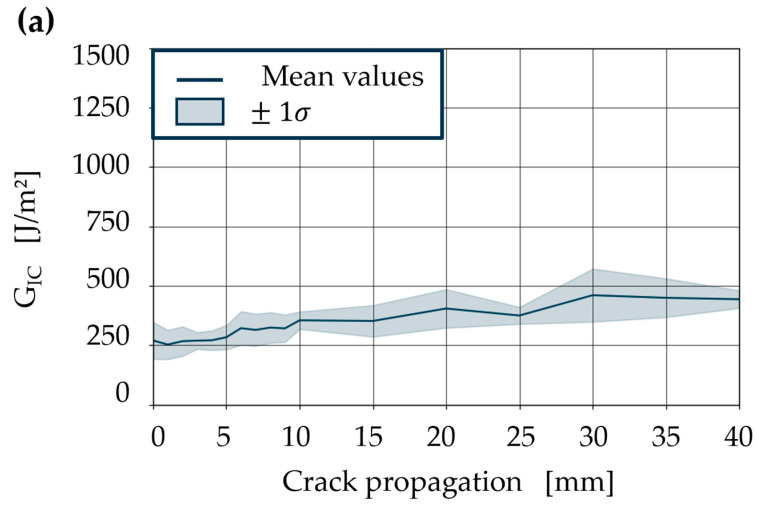

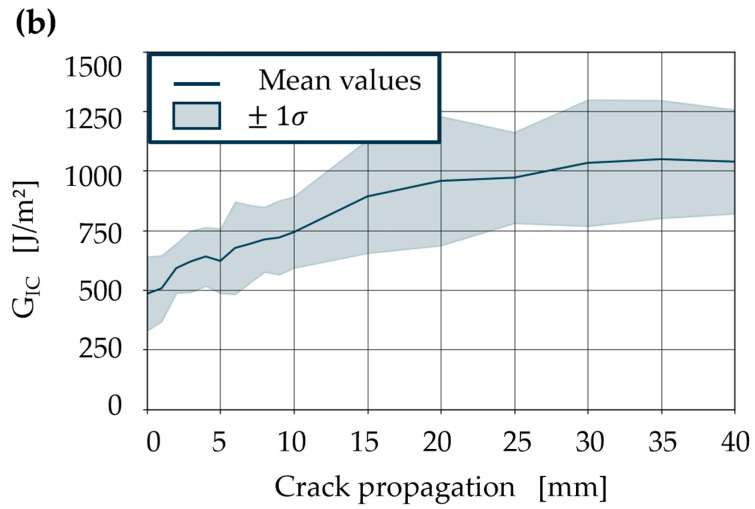
*G_IC_* of epoxy resin (**a**) and vitrimeric resin (**b**) at 120 °C.

The comparison of the *G_IC_* of the CFRV with that of the reference epoxy at 120 °C, above T_v_, reveals roughly double *G_IC_* for the vitrimer resin (Figure 4). The reference sample demonstrates a constant *G_IC_* with a reduced standard deviation, while the vitrimer sample exhibits elevated energy absorption during crack propagation, accompanied by a higher standard deviation. The same behaviour can be demonstrated for the required energy for crack initiation. The reference epoxy resin shows approximately 224 J/m^2^ as the energy needed for crack initiation, whereas for the vitrimer resin, this figure is 505 J/m^2^. This discrepancy must be attributed to the CAN, since epoxy delamination is brittle and shows little or no plastic deformation at this temperature. When the disulfide bonds rearrange above T_v_, in vitrified samples, a higher *G_IC_* can be observed due to the enlargement of the fracture process zone as a result of decreasing yield strength and viscoelastic dissipation mechanisms, particularly in advanced crack fronts with values above 1000 J/m^2^. This process can promote fibre bridging or the release of fibres from the matrix. The aforementioned effects result in an increase in the *G_IC_* and are discussed in detail.

**Figure 5:**
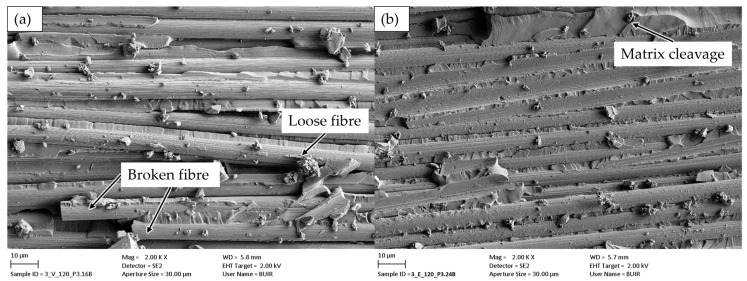
FESEM images of CFRV first-generation specimen detached at 120 °C (**a**) and epoxy resin detached at 120 °C (**b**).

Evidence of differences in G_Ic_ is apparent upon microscopic examination of the first-generation specimen. The crack surfaces displayed in Figure 5 offer a comparative analysis of vitrimer specimens and first-generation epoxy specimens that have been detached in the initial generation from the area of crack propagation. In addition to the ripple effect, which is indicative of brittle failure, the vitrimer displays a significant prevalence of loose and broken fibres, suggesting the occurrence of fibre pull-out or fibre bridging in the fracture process zone (Figure 5a). Conversely, the smooth surface of the epoxy only permits conclusions regarding brittle detachment even at elevated temperatures. Additionally, the images suggest that the vitrimer exhibits adequate fibre–matrix adhesion. The individual fibres are pulled out of the fracture plane in conjunction with matrix residues. Furthermore, the presence of numerous completely encapsulated fibres is evident in Figure 5a.

### 3.2. Temperature Influence on Vitrimer Detachability

**Figure 6:**
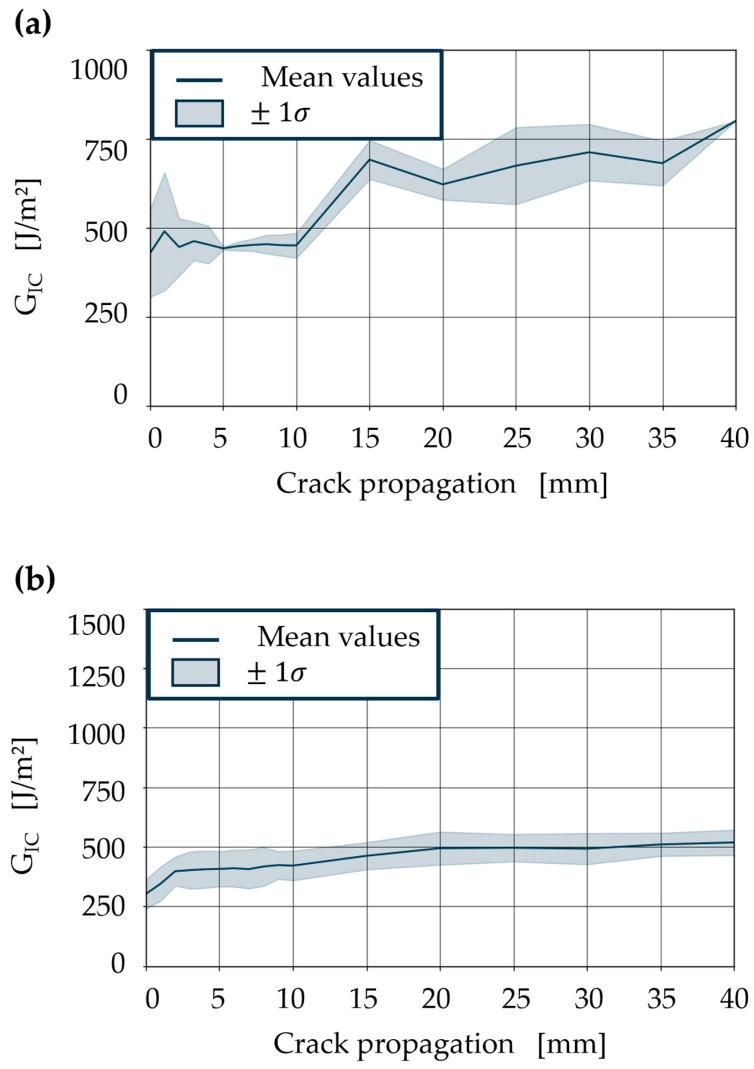
*G_IC_* of vitrimeric resin first-generation specimen, comparing room temperature (**a**) with elevated test temperature at 80 °C (**b**).

Below T_v_, the *G_IC_* while detaching the specimen with a vitrimer matrix decreases (Figure 6). Due to the crack induced by the PTFE film in the experiment, crack initiation at room temperature (364 J/m^2^) is only slightly higher than at 80 °C (326 J/m^2^). The local maxima of the crack propagation in Figure 6a show that the vitrimer matrix allows for non-homogeneous energy dissipation along the front of the crack at room temperature. This can be explained by crack delay, crack jumps, and heterogeneity within the material. As the test temperature increases, the stiffness of the material decreases and its viscoelastic relaxation capacity increases. This should enable the vitrimer to adapt to local inhomogeneities. On the other hand, the *G_IC_* required for crack propagation should decrease due to the lower stiffness. This effect can be seen when comparing *G_IC_* at 80 °C with room temperature tests. Effects such as fibre bridging and crack delay occur significantly less frequently. This allows the crack front to spread continuously along the intermediate layer in the laminate. This can be seen in the more continuous and stable energy release rate.

Comparing these results with a test temperature above T_v_, where the CAN is activated, shows a direct effect on the *G_IC_* curve, as seen in Figure 4b. At this temperature, effects such as fibre bridges, fibre pull-out, microcrack formation, plastic and viscoelastic deformation of the matrix, and the resulting friction and sliding processes contribute more significantly to the *G_IC_*. The fracture process zone (FPZ) around the crack enlarges, thereby increasing the contribution to energy dissipation before the crack can grow.

### 3.3. Detachability of Multiple Composite Generations

**Figure 7:**
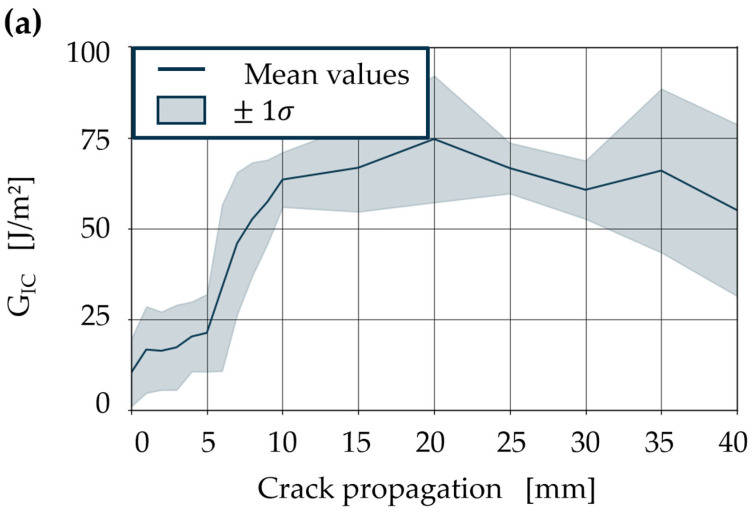

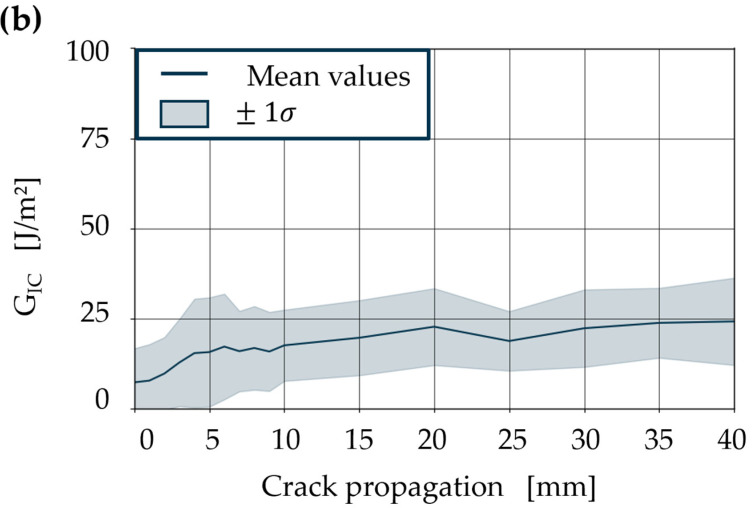
*G_IC_* of vitrimeric resin second-generation specimen at 80 °C (**a**) and 120 °C (**b**).

The *G_IC_* values for the second-generation samples produced with the vitrimer matrix are 10 to 20 times lower compared to those of the first generation (Figure 7). Visually, the samples appear seamlessly reconnected, with no obvious manufacturing defects after 45 min of reconsolidation at 190 °C with approximately 8.5 MPa. The samples were separated at the same temperatures as the first-generation specimen. The lower *G_IC_* can be explained by the crack tearing along the energetically weak interface after reaching its maximum. The causes for the lower energy release rates of the reconnected samples are manifold in nature. On the one hand, polymer diffusion can be limited at the interfaces even at high temperatures. On the other hand, effects such as surface glazing due to oxidation of the polymer chains on the surfaces of the laminate can reduce reactivity. In addition, the topography of the separated surfaces plays a decisive role in reconnection. Unevenness or a ‘smooth’ surface leads to partial contact, which also results in a weaker connection in terms of energy. This is why the material degradation, especially in terms of surface roughness, needs to be evaluated.

**Figure 8:**
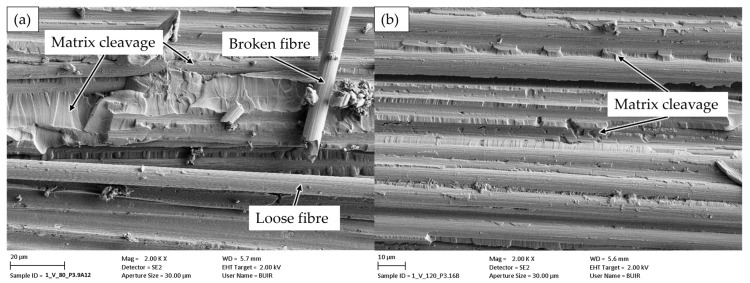
FESEM image of fractured surface from second-generation CFRV detached at 80 °C (**a**) and 120 °C (**b**).

A comparison of the reconsolidated and detached CFRV samples from the crack initiation zone indicates that at the lower temperature of 80 °C, slightly more loose fibres are present in the fracture process zone, which would result in a higher recorded G_Ic_ (Figure 8). At 120 °C, the specimen exhibits matrix cleavage that is comparable to the brittle-dominated demoulding of the epoxy sample (compared in Figure 5). Overall, the introduction of a higher error count in the laminate was previously described by Zhao et al. [22]. A general observation of the fracture patterns recorded for the second generation reveals that these are somewhat smoother on the surface, which is considered in Section 3.2 and could argue against proper reconsolidation. However, it is evident that there remains sufficient fibre–matrix adhesion and proper impregnation in the second generation, as observable in resin-rich regions in the SEM images.

As an interim conclusion of the DCB test, it can be stated that a higher *G_IC_* is required for the detaching of CFRVs, particularly at temperatures above T_v_. However, this does not indicate poorer recyclability but rather demonstrates the unique behaviour of the vitrimeric resin. Although the *G_IC_* values determined for the second-generation specimen are significantly lower, this may be partly due to underestimation by the used loading cell with minimum force recorded at 10 N. Micrographs of the reconsolidated cross-section show a completely healed crack with no visually detectable defects. To further evaluate the recyclability of endless CFRVs, the surface area of the detached laminate layers is analysed to indicate the degradation of the composite as well as correlate them with the observed *G_IC_*, which occurs due to material breaking or a limited amount of time for adaptive networks to debond during detaching.

### 3.4. Detachment Mechanisms Visualised by Surface Roughness

High-precision tactile measurement of the surface of the separated areas allows for the observation of mechanical and thermal degradation. The increase in surface roughness can be attributed to various properties of the DCB test: covalent bonds are detached when the layers are separated, and these matrix cracks, which expand parallel to the crack propagation, are measurable on the surface. The tearing of fibres along the interlaminar layer causes a fibre-dominated increase in surface roughness. Ultimately, a detachment of single fibres due to the release of fibre–matrix adhesion can be measured.

**Figure 9:**
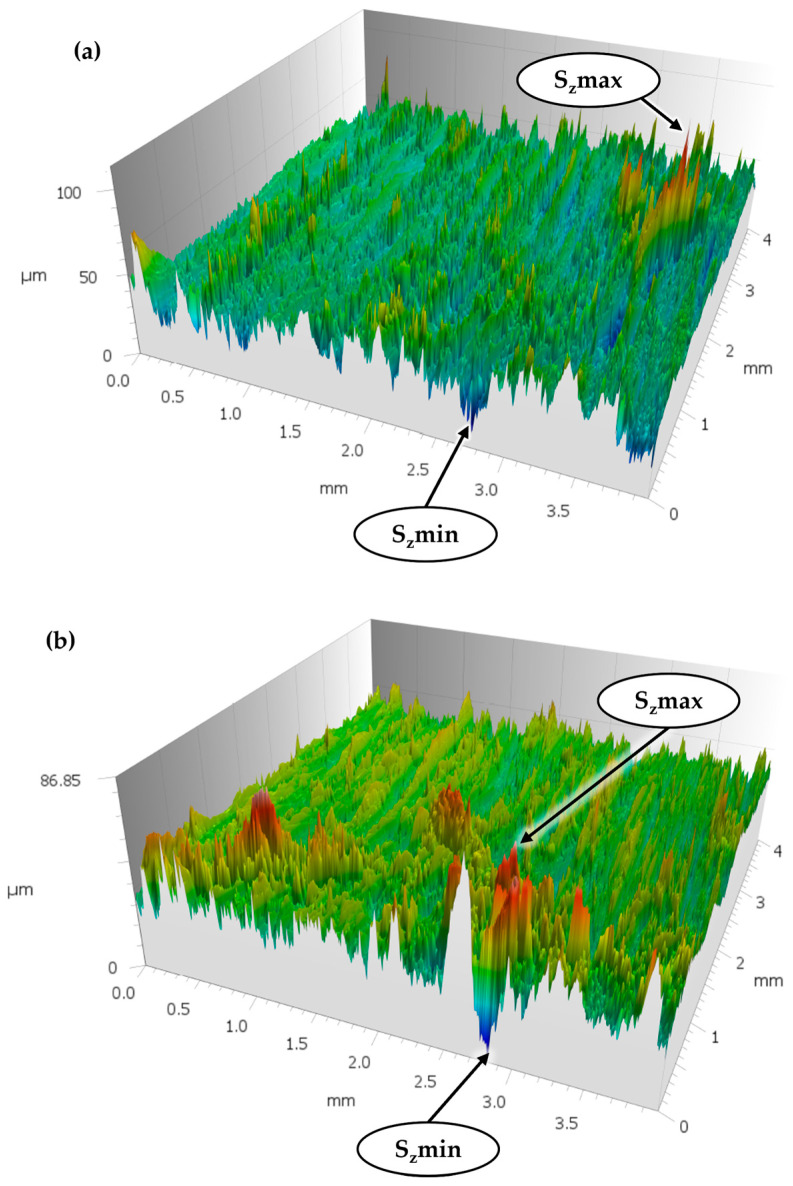
Surface roughness of CFRV tested at 120 °C, including its first generation (**a**) and second generation (**b**).

It is crucial to make a direct surface comparison between iterations of the specimen generations. This enables an analysis of the changes that occurred after the first and second separations. The extremes (S_z_min and S_z_max) demonstrate the absolute differences between surface profiles of the CFRV specimen. The ridges on the surface follow the fibre orientation, while the tactile sensor is moved perpendicular to it (Figure 9). S_z_min is located in the same location of the measured surface area, which is not unusual, as local extrema tend to allow for poorer reconsolidation properties. Therefore, these extrema are much more pronounced in the second-generation sample.

**Figure 10:**
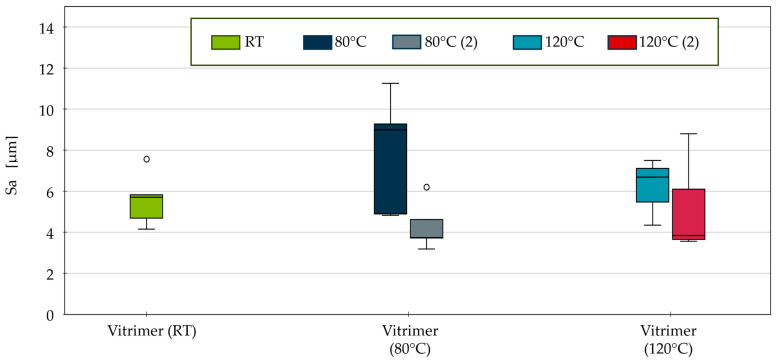
Arithmetic mean surface roughness of all tested specimens of first generation and second generation marked with (2) after DCB.

The general arithmetic mean surface roughness indicates that the detachment mechanisms can be observed for CFRVs after separation, especially following the initial detachment at elevated temperatures (Figure 10). These values range from a median of 5.8 µm at room temperature to a maximum median of 9.2 µm at 80 °C. While the surface roughness of the epoxy samples remain within a constant range regardless of temperature, those of the vitrimers are significantly more inhomogeneous and temperature-dependent, exhibiting varying surface roughness values.

At 80 °C, the dynamic bonds are inactive, which is why there is no increase in the interlaminar energy release rate. At the same time, in contrast to regular epoxy resin, the network in this temperature range has slight plastic deformability. In this temperature range, the crack propagates mainly through brittle-dominated sections, supplemented by isolated viscoelastic relaxation zones, shown by closely localised S_z_min and S_z_max. In addition, the loads are absorbed more strongly by the fibres in the samples of the first generation. This leads to a higher probability of fibre pull-out and fibre fragmentation. When examining the individual areas, this can be seen in the form of fragments, which appear as peaks, whereas this is largely absent in samples from the second generation.

A comparison of the results for the second generation reveals that the surface roughness has reduced to the same level as the reference epoxy samples, which averages between 3.4 µm at room temperature and 4.7 µm at 120 °C. The CFRV demonstrates two parallel effects. Firstly, hot pressing the samples significantly reduces the surface roughness in the interlaminar layers at 190 °C. At this temperature, the self-healing effects of the vitrimer matrix are activated. Additionally, the *G_IC_* decreases significantly in the second DCB run, generally indicating less fibre- and matrix-dominated damage to the interlayer. Here, the self-healing effect at 190 °C dominates, as samples from the first generation that were particularly damaged at 80 °C now show lower surface roughness on average.

**Figure 11:**
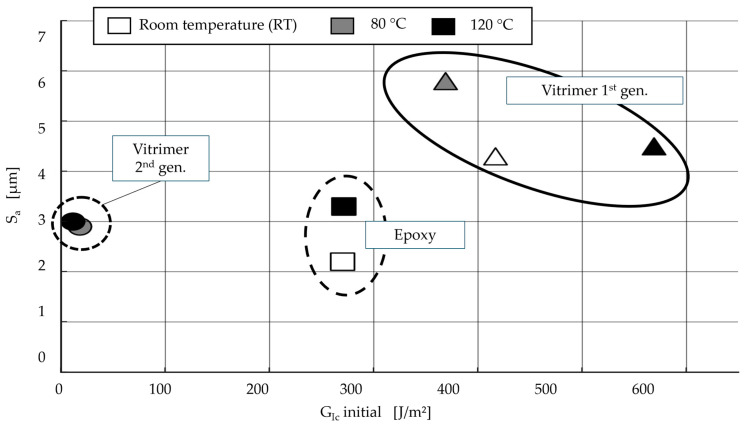
Surface roughness of dependency of *G_IC_* for different temperatures with three distinct groups of CFRV, including first generation (triangle), second generation (circle), and epoxy (square).

Figure 11 shows the *G_IC_* required for detachment plotted against the measured surface roughness. Three distinct groups can be identified, which underline the effects described in this study. At high *G_IC_*, the average surface roughness increases, as can be seen in the group of first-generation vitrimers. The brittle fracture behaviour in combination with the medium measured *G_IC_* of the epoxy samples produces lower surface roughness in the fractured surface. These data points are closer together because the temperature influence on the epoxy is lesser. The second-generation vitrimer samples also show lower *G_IC_* values overall and no significant differences in surface roughness.

Overall, it can be seen that damage to the unidirectional CFRV can be tactilely measured and that the influence of recycling can be evaluated. An examination of the individual areas can reveal detachment mechanisms such as fibre pull-out or matrix cracks along the crack surface. However, the effects of higher surface roughness can be levelled out by the self-healing properties of the vitrimer.

## 4. Conclusions

Thanks to their CAN, endless CFRVs offer a promising opportunity to enable circular composite strategies. Reattached samples of CFRVs have been shown to be able to detach and reconnect, using a hot-pressing process without visible defects in the zone of reconsolidation. The *G_IC_* of vitrimeric resin, determined via DCB, shows that detaching above T_v_ produces a higher *G_IC_*, which is significantly higher than *G_IC_* for epoxy resin. Unfortunately, due to the reduction in stiffness, it was not possible to determine a value above T_g_ for the vitrimer matrix, which as expected, should enable significantly lower *G_IC_* values.

The influence of detachment on the surface roughness of the laminate was higher for the first-generation CFRV than for the reference samples due to the higher *G_IC_*. The second-generation specimen exhibited a significantly lower *G_IC_*. Surface roughness after detaching was also lower. While the CFRV is recyclable, vitrimer-specific properties above T_v_ and, in particular, above T_g_, must be considered in the process design to reuse the material efficiently. However, the material’s self-healing property is a decisive advantage, as it can reduce damage. This potential could be demonstrated in further research by incorporating virgin material in the reconsolidation area for better self-healing ability during hot pressing. With this adaptation, the reuse of endless CFRVs can be established in the future.

## Data Availability

The original contributions presented in this study were included in the article. Further inquiries can be directed to the corresponding author.
